# Prevalence and determinants of exclusive breastfeeding among adolescent mothers from Quito, Ecuador: a cross-sectional study

**DOI:** 10.1186/s13006-015-0058-1

**Published:** 2015-12-10

**Authors:** Miguel Á. Jara-Palacios, Angélica C. Cornejo, Gabriela A. Peláez, Jenny Verdesoto, Andrés A. Galvis

**Affiliations:** Escuela de Medicina, Facultad de Ciencias Médicas de la Salud y de la Vida, Universidad Internacional del Ecuador, Quito, Ecuador; Escuela de Ciencias Físicas y Matemáticas, Universidad de las Américas, Quito, Ecuador

**Keywords:** Exclusive breastfeeding, Adolescent mothers, Quito, Ecuador

## Abstract

**Background:**

Exclusive breastfeeding (EBF) is the optimal way to feed children during their first six months of life, having important benefits for them and their mothers. However, the proportion of Ecuadorian mothers who continue to exclusively breastfeed their infants during the recommended six-month period has been reported to remain below the World Health Organization’s goal set of 90 %. Little is known regarding factors influencing adolescent mothers to decide whether to practice EBF or not. Furthermore, there is no data about the EBF rates among adolescent mothers in Quito, Ecuador.

**Methods:**

This cross-sectional study took place from April to November 2013 in the largest maternity ward in Quito, Ecuador (*Hospital Gineco Obstétrico Isidro Ayora*). Adolescent mothers parenting an infant between 6 and 24 months of age (n = 375) were interviewed using a structured questionnaire about EBF knowledge, beliefs and practices. Bivariate and multivariate analyses were used to identify the independent predictors of EBF.

**Results:**

In our sample, 62.9 % of adolescent mothers raising infants between 6 to 24 months of age chose EBF. Knowledge about the maternal benefits of breastfeeding and awareness of appropriate time frame for EBF were statistically associated with increasing the likelihood of choosing EBF. Adolescent mothers who were acquainted with the recommended duration of EBF were more likely to practice EBF (adjusted odds ratio (AOR) = 1.73; 95 % confidence interval (CI) 1.003, 2.98) as well as those who knew that breastfeeding is a protective factor against breast cancer (AOR = 5.40; 95 % CI 1.19, 24.56).

**Conclusions:**

Although adolescent mothers may be more prone to discontinuing EBF before their infants reach six months of age, the prevalence of EBF among adolescent mothers interviewed was higher than the rate reported for Ecuadorian mothers in other age groups. Our data underscores the importance of emphasizing the correct practice of BF and its benefits in breastfeeding education programs provided to Ecuadorian mothers, in order to promote the extension of breastfeeding duration to the recommended levels.

## Background

Exclusive breastfeeding (EBF) of infants during their first six months of life is crucial to promote growth and development. Continuous breastfeeding complemented with additional food sources is recommended until the second year of life [1]. Breastfeeding is the optimal way to feed newborns [1] having important benefits for mothers and their infants. Major infant benefits include: decreased risk of childhood infections [2–4], reduced postnatal mortality rates [4], decreased sudden infant death syndrome rates [5], lowered probabilities of developing diabetes [6], improved cognitive and motor development [7], among others. Maternal breastfeeding benefits include: lower risk of developing breast and ovarian cancers [2], adequate weight recovery [8] and lactational amenorrhea which could be a natural birth control [2, 3].

For all these reasons, breastfeeding is a public health priority promoted and supported by “The Global Strategy on Infant Young Child Feeding” approved by the 55th World Health Assembly (WHA) in 2003 [1]. Breastfeeding in Ecuador constitutes a natural right for infants according to the “Law for Promotion, Support and Protection of Breastfeeding” (Ley de Fomento, Apoyo y Protección a la Lactancia Materna), approved in 1995. Furthermore, since 2009, the commercialization and marketing of breast milk substitutes has been regulated in order to promote the International Code of Marketing of Breast-milk Substitutes [9]. However, the EBF rate in the Ecuadorian population is 43.8 % at six months according to the “National Survey of National Health and Nutrition” (“Encuesta Nacional de Salud y Nutrición”, ENSANUT) published in 2013 [10]. This survey does not provide specific information for adolescent mothers. However, a 2004 study stated that adolescent mothers tend to have lower EBF rates than the general population [11]. Lower rates of EBF among adolescent mothers may be explained by factors such as lower income and education [12, 13], higher percentage of single mothers in comparison with older women [13], cultural beliefs [14] and lack of information about breastfeeding [15].

Breastfeeding duration among adolescent mothers may be associated with demographic characteristics such as educational level and multiple births [12, 16, 17]. Mothers with less education tend to introduce other foods earlier, in contrast to mothers who have higher education level [16]. This may be related to increased access of information and recognition of the benefits of exclusive breastfeeding for mothers with higher education [12, 16]. Additionally, previous pregnancies are associated with higher likelihood of adolescent mothers choosing EBF [17]. A higher likelihood of EBF among this group of mothers has also been associated with counselling sessions [18], unemployment [19], supportive family and peers, cultural acceptance, maternal beliefs regarding breastfeeding and health of the infant, previous successful breastfeeding experience, workplace support, among other factors [20].

The Pan American Health Organization (PAHO) defines adolescence as the period between 10 and 19 years of age [21]. In Ecuador, adolescent mothers are responsible for about 20 % of the total reported births [22]. Therefore, a further understanding of the determinants of EBF practice among adolescent mothers is necessary to develop policies that will improve breastfeeding rates and allow reaching the World Health Organization goal of 90 % EBF at six months [23]. This study aimed to estimate the prevalence of adolescent mothers in Isidro Ayora’s Hospital (the largest obstetrics and gynecology hospital in Quito, Ecuador’s capital city), who exclusively breastfeed their infant under six months of age and to identify the main factors influencing their decision to practice EBF.

## Methods

### Study setting and participants

A cross sectional study was conducted between April and November 2013 in the Hospital Gineco Obstétrico Isidro Ayora (HGOIA) in Quito, Metropolitan district, Ecuador. The inclusion criteria were adolescent mothers (10 to 19 years old) raising children aged 6 to 24 months who were living in Quito, Ecuador at the time of the study and who sought child health check-up for their infants at the Adolescent Area of Isidro Ayora’s hospital. The only exclusion criterion was the mother’s refusal to participate in the study.

Quito is the capital of Ecuador, located in the Andean region with a population of about 2 million [24]. The Ecuadorian Health System is formed by public and private institutions. The Ministry of Public Health (Ministerio de Salud Pública- MSP) manages the public health institutions which provide health care services to the entire population. Patient care in public hospitals is free during pregnancy, childbirth and postpartum [25]. The HGOIA is the biggest public maternity ward in Quito; where approximately 10,000 births are attended per year and over 30 % correspond to adolescents [26].

The survey instrument was designed and validated by a different group of adolescent mothers to ensure comprehensibility of each question. The questionnaire included information about demographic characteristics of participants, personal breastfeeding practices, knowledge, beliefs, and previous counseling experience about breastfeeding. These questions were postulated in the order mentioned above and based on the factors that other studies have found to be important in determining EBF practice [1, 12–17]. The sample size was determined using a formula for estimation of single population proportion [27] using the expected prevalence for EBF of 39 % which had been previously reported [11], with a 95 % confidence level, and a 5 % margin of error. A total of 390 mothers were interviewed in HGOIA’s adolescent outpatient service. The responses of 375 mothers were included in the analysis. Fifteen surveys were excluded due to errors during data collection and processing. A written informed consent, approved by the Bioethics Committee of the International University of Ecuador, was obtained for all study participants. The number assigned for this study by the Bioethics Committee was 02-2013.

### Measurements

Maternal sociodemographic and obstetric variables recorded were: age, educational level, ethnicity, place of birth and residence, religion, marital status, occupation (working, not working, studying), place of delivery, number of prenatal control visits, multiple or single pregnancy, gestational age at birth and delivery method. Infant variables registered were gestational age at birth and age at the time of the interview.

We assessed breastfeeding prevalence, initiation and duration. EBF was defined as the infant having received only breast milk during the first six months of life (including expressed milk or from a wet nurse) and no other liquids or solids except medicines, oral rehydration formulas, vitamins and minerals. When EBF was not provided, the age at which additional or alternate food was introduced in the infant’s diet, the type of food added and the reasons for discontinuing EBF were recorded. In order to verify early skin-to-skin contact, mothers were asked about the time elapsed between delivery and first breastfeed.

To study perceptions regarding breastfeeding, mothers were asked about their perceived benefits of breastfeeding for their child. Subsequently, they were given options about potential advantages that breast milk could have on infants in order for them to select the most likely ones to be correct. Additionally, they were asked about their perception of the effects of breastfeeding on themselves.

To evaluate their knowledge about breastfeeding, the interviewees were asked about the recommended length of the EBF, the duration of each feed and the period between each one. They were also asked if they considered maternal milk superior to infant formula or vice versa, and finally, if they received breastfeeding counseling during their stay at the maternal center they delivered in.

### Data processing and analysis

The data were analyzed using Statistical Package for the Social Science (SPSS) software for windows 20.0. Chi-square test was used to evaluate the association between EBF prevalence and each variable. The odds ratios with 95 % confidence intervals were calculated in order to evaluate the risk of independent variables; variables with *p* < 0.05 (knowledge about the recommended time of EBF and awareness that EBF helps to prevent breast cancer) and variables that are commonly linked with the practice of EBF (occupation, marital status, counselling regarding EBF and type of delivery) [12–19] were used in the multivariate logistic regression model in order to determine the strength of association between these variables and EBF.

## Results

### Sample characteristics

A total of 375 adolescent mothers were included in this study. The mean age (standard deviation) of the mothers was 17.33 (±1.2) years, with a range from 14 to 19 years. The majority of women were married/cohabiting (55.7 %) and had at least completed the first year of secondary school (82.9 %). Housewives made up 45.1 % of all mothers, 38.9 % continued studying after delivery and 4.8 % started college (Table 1).

**Table 1:** Socio-demographic data of interviewed adolescent mothers

Characteristics	Frequency	Percent
Age (years)		
14–16	88	23.47
17–19	277	76.53
Education		
None	3	0.80
Primary	43	11.47
Secondary	311	82.93
College	18	4.80
Marital Status		
Single/divorced	166	44.27
Cohabiting/Married	209	55.73
Occupation		
Employed	60	16.00
Studying	146	38.93
Housewives	169	45.07
Religion		
Catholic	207	55.20
Evangelist	56	14.93
Christian	18	4.80
Others	6	1.60
None	88	14.93

### Exclusive breastfeeding rate

About 98.7 % of the women interviewed in this study, stated having initiated breastfeeding within the first days of the baby’s life. However, 88.0 % of them continued EBF after the baby reached the first month of age. This percentage continued to decrease over time; 80.8 % of the mothers decided to continue this activity three months after delivery. By the month six after birth, 62.9 % of the infants were being nourished with breast milk exclusively. The mothers who fed their child with other types of liquid or food besides breast milk before age six months, introduced formula (45.2 %), 25.6 % received “coladas” (a beverage made out of water and any kind of cereal flour), water (17.1 %) and other food (12.1 %) to their infant’s diet. It is important to indicate that 14.1 % of the participants used milk from another woman at least once, whether from a wet nurse or from milk banks.

Nearly 56.5 % of the interviewees knew the recommended duration of EBF. Women who were aware of the appropriate duration of EBF were more likely to exclusively breastfeed their babies (68.4 %) compared to those who were unaware of the recommended time (55.8 %, *p* < 0.05), showing that knowledge of the recommended duration of EBF increases the likelihood of practicing 1.7 fold (Table 2).

**Table 2:** Association between EBF rates with socio-demographic factors and health services related factors among 375 participants

	Total (N)	EBF (N)	EBF (%)	OR (95 % CI)	*p* value
Mother					
Age					
14–16	88	49	55.7	1	
17–19	277	187	65.2	1.49 (0.9, 2.4)	0.130
Education					
None/primary	46	25	54.3	1	
Secondary/college	329	211	64.1	1.50 (0.8, 2.8)	0.254
Marital status					
Single/divorced	166	105	63.3	1	
Cohabiting/married	209	131	62.7	0.98 (0.6, 1.5)	0.915
Religion					
Catholic	207	133	64.3	1	
Other or none religion	168	103	61.3	1.13 (0.7, 1.7)	0.592
Occupation					
Employed/Studying	195	122	62.6	1	
Housewives	180	114	63.3	0.97 (0.6, 1.5)	0.915
Number of prenatal consultations					
1–4	50	32	64.0	1	
5 or more	325	204	62.8	0.95 (0.5, 1.8)	1.000
Birth					
Vaginal	288	189	65.6	1	
Cesarean	87	47	54.1	1.62 (1.0, 2.6)	0.058
Initiation of breastfeeding					
Within the first hour after birth	46	24	52.2	1	
After the first hour after birth	329	212	64.4	0.60 (0.3, 1.1)	0.142
Counselled about breastfeeding					
Yes	282	177	62.8	1	
No	93	59	63.4	0.97 (0.6, 1.6)	1.000
Knowledge about the recommended time of EBF					
Yes	212	145	68.4	1	
No	163	91	55.8	1.71 (1.1, 2.6)	0.013
Know that breastfeeding helps to prevent breast cancer					
Yes	17	15	88.2	1	
No	358	221	61.7	4.65 (1.0, 20.6)	0.037
Children’s information^a^					
Gestational age (GA) at birth					
Full term	265	169	63.8	1	
Preterm or post term	103	62	60.2	1.16 (0.7, 1.9)	0.549

^a^Some mothers did not know the GA at birth

Although only 4.5 % of interviewees were aware that breastfeeding can reduce the probability of developing breast cancer in the future, the majority of them (88.2 %) practiced EBF until the infant reached six months of age. Mothers who were not familiarized with this maternal benefit, did not show such a high percentage of EBF practice (61.7 %). Therefore, the mothers who knew that breastfeeding is a protective factor against breast cancer are 4.6 times more likely to EBF their infants (Table 2).

Adjusted odds ratio (AOR) shows that mothers who knew the recommended time of EBF, presented higher odds for EBF than those who were unaware of this recommendation (AOR = 1.73; 95 % CI 1.003, 2.98). Likewise, the odds of EBF were higher if mothers knew that breastfeeding helps prevent breast cancer (AOR = 5.40; 95 % CI 1.19, 24.56) (Table 3).

**Table 3:** Multivariate logistic regression model of the predictors of exclusive breastfeeding

Variable	AOR^a^	95 % CI	*p*-value
Knowledge of EBF definition			
No	Reference		
Yes	1.73	1.003, 2.98	0.049
Know that breastfeeding helps to prevent breast cancer			
No	Reference		
Yes	5.40	1.19, 24.56	0.029

*AOR* adjusted odds ratio

^a^Variables included in the model: Occupation, marital status, advice about breastfeeding, type of birth, knowledge the meaning of EBF, know that breastfeeding helps to prevent breast cancer

### Mothers’ reasons for suspending EBF

The majority of adolescent mothers in this study breastfed their newborns at least once. Five women reported not producing milk and thus never breastfeeding their infants.

Among the 136 participants who initiated breastfeeding but introduced another food source before the infant reached sixth months of age, the most common causes for stopping EBF included: insufficient breast milk (26.3 %), the necessity of returning to school or go to work (21.7 %), maternal choice (14.5 %), medical indication (13.8 %) and “other reasons” (8.6 %) (Table 4). Other explanations for suspending EBF included: infant disease, inadequate quality of breast milk, absence of suction from the infant, nipple pain and pre-term delivery.

**Table 4:** Reasons for not initiating or stopping breastfeeding among adolescent mothers in Quito

	Yes	(%)
Reasons for not initiating breastfeeding		
I never had milk	4	(80.0)
Use of anticonvulsants	1	(20.0)
Reasons for discontinuing EBF^a^		
Insufficient milk	40	(26.3)
Othere occupations (study, work, etc.)	33	(21.7)
Maternal choice	22	(14.5)
Medical indication	21	(13.8)
Other	13	(8.6)
Maternal disease	9	(5.9)
Family or friends advice	7	(4.6)
Natural weaning	7	(4.6)

^a^Some of mothers gave more than one reason for discontinuing EBF

### Beliefs about breastfeeding

Over 96.5 % of women thought breast milk was better nourishment for their infants than infant formula. There were no demographic differences between this group of mothers and the 3.5 % of them who did not think breast milk is the best nutritional choice for their infants. Adolescent mothers believed breast milk offered several benefits for their babies, such as: helping them to have adequate weight (93.3 %) and height (86.9 %), improving intellectual development (77.3 %), creating a link between mother and child (76.8 %), preventing future infections (76.3 %) and improving psychomotor development (71.5 %) (Table 5).

**Table 5:** Adolescent mothers’ beliefs related to effects of breastfeeding on the child

	Agree n (%)
Infant formula is better than breast milk	13 (3.5)
Breastfeeding helps babies to have adequate weight	350 (93.3)
Breastfeeding helps babies to have adequate height	326 (86.9)
Breastfeeding improves intellectual development	290 (77.3)
Breastfeeding establish a link between mother and child	288 (76.8)
Breastfeeding helps to prevent future infections	286 (76.3)
Breastfeeding improves psychomotor development	268 (71.5)
Breastfeeding helps to prevent allergies	210 (56.0)
Breastfeeding improves social child abilities	209 (55.7)
Breastfeeding helps to prevent chronic disease at adulthood	203 (54.1)

## Discussion

Usually, an adolescent mother is considered less likely to continue EBF in comparison to older women due to the higher likelihood of young women being single [13], having low educational levels and lower income [12, 13] and due to the urgency of attending school [28]. Nevertheless, this study showed no strong relationship between those factors and the cessation of EBF before age of sixth months. Therefore, as proposed by Sipsma and collaborators, enhanced clinical support and promotion of EBF, may be more influential than interventions aimed at improving the above mentioned factors [29].

This study showed that about 62.9 % of adolescent mothers delivering at the Isidro Ayora’s hospital, exclusively breastfeed their infants within the first six months of life. This rate is higher than the one estimated for all mothers in Ecuador (43.8 %) [10] and higher than the EBF prevalence reported in other countries among adolescent mothers (which has been reported to range from 52 % in the United States of America to 13.8 % in Brazil) [13, 14, 18]. There are several possible explanations for these findings. First, adolescent mothers are less likely to be employed [19] and we found a high rate of mothers who left school after or during pregnancy, therefore, they have more opportunity to stay at home taking care of the infant, including EBF. Second, although adolescent mothers have lower education levels [12, 17], the majority of surveyed mothers knew the recommended time of EBF and agreed that breast milk is the best food source for their infants; as a result, those may be a motivation for them to EBF their infants [30]. Finally, HGOIA provides free maternity services [25, 26]; for this reason, mothers who attend to this center could be more likely to have lower incomes, so nourishing their babies with breast milk exclusively may be economically more convenient than buying infant formula.

Although the definition of EBF includes breastmilk from a wet nurse, there are different implications when the infant is not fed by their mothers. La Leche League discourages this practice for several reasons; the most important ones are the risk for the baby of acquiring an infection and being affected by chemical contaminants such as drugs that may be used by the wet nurse. Also, the milk from another mother, whose infant does not have the same age, may not provide the components the other baby needs according to his age [31]. Therefore, human milk provided by an adequately managed human milk bank is the best option if the mother is unable to express her own milk [32]. Our results show that approximately 14 % of all mothers used milk from a donor. Considering that it is a high percentage, we recommend that the risks, indications and precaution for using human milk from a donor should be part of the breastfeeding counseling.

On this study, the prevalence of EBF was higher in the group of mothers who knew the recommended length of time for EBF. Our results are in agreement with previous studies reporting the association between the awareness of the WHO recommendations with initiation and duration of EBF [33]. According to Renfrew and collaborators, the breastfeeding guidance provided personally by trained staff and given during an accessible schedule, increases the duration of EBF [34]. However, adolescent mothers who denied having received breastfeeding assistance did not present a higher rate of early discontinuation of EBF.

The results show that less than 5 % of all participants know that breastfeeding helps prevent breast cancer. The lack of awareness about the benefits of breastfeeding to the mother’s health has been previously reported [35]. Hence, there is a necessity to promote the mother’s knowledge about maternal benefits of breastfeeding in order to optimize breastfeeding initiation and maintenance.

One of the most important factors affecting the adolescent mother’s decision to breastfeed their babies is the family’s and partners’ support. On this study, 55 % of adolescent mothers were married or cohabiting and, some of them lived with multiple immediate or extended family members that could have influenced their EBF decision [36–38]. Unfortunately, there is no data that determine neither the family members’ role nor the partners’ role in the mother’s breastfeeding behaviors. Therefore, although there was no significant relationship between marital status and the mother’s breastfeeding behaviors, we consider this point as an avenue for future research.

Regarding the reasons given by mothers for early discontinuation of EBF, this study highlights the perception of low breast milk production. This result is similar to the one previously reported in Ecuador, establishing that this reason prevailed as an important consideration in the duration of breastfeeding [39]. However, only about 5 % of all mothers are not physiologically able to produce enough milk, so the real cause of insufficient milk intake is the inability of the infant to extract milk, due to inappropriate early feeding routines [40]. This means that the perception of low milk production can be generally avoided by teaching the mothers about the correct way to feed their babies.

Our study is limited to mothers who were attended the maternity hospital in which a breastfeeding counseling program is freely available. In addition, all the participants involved in the study came from an urban area where the population has a higher education level, higher incomes and a lower fertility rate than the rural population [10]. Therefore, the EBF prevalence may be dissimilar from other samples of mothers in Ecuador. Furthermore, our study is based on self-reporting by the mothers, and the questions asked to them were posed in a specific order with the aim of obtaining sincere answers, particularly in the question about the baby’s age in which EBF was suspended. Despite this fact, mothers might have felt compelled to respond according to the information they received during counseling (whether or not they truly followed the guidelines), since the interviewers were always health personnel, wearing medical coats.

## Conclusions

Contrary to what we expected, based on previous studies; the prevalence of EBF among the sample of adolescent-mothers was higher than that reported for the general population of mothers in Ecuador. In order to verify and explore in depth this unexpected high rate of EBF, there is a need for future research among a sample of adolescent mothers with greater generalizability. Regarding the determinants of EBF among adolescent mothers; the results of this study suggest that in order to increase the likelihood of EBF practice, health care providers should implement or increase the information about the maternal benefits of breastfeeding, as well as the time indicated for EBF. This knowledge might be generalizable to other countries in the region and should be studied as a venue to promote breastfeeding in promotion programs in other low and middle income countries, especially those culturally and socially similar to Ecuador, such as those in the Andean region (Colombia, Peru, Bolivia). Furthermore, additional research is needed on how knowledge of breastfeeding influence in the duration of this practice. Finally, as adolescent mothers deal with multiple barriers to achieve a successful breastfeeding, we encourage developing guides for breastfeeding policies specifically for this population considering our results and limitations.

## Abbreviations

EBF: Exclusive breastfeeding
HGOIA: Hospital Gineco-Obstétrico Isidro Ayora
PAHO: Pan American Health Organization
WHO: World Health Organization
